# Patients without colonoscopic follow-up after abnormal fecal immunochemical tests are often unaware of the abnormal result and report several barriers to colonoscopy

**DOI:** 10.1186/s12876-020-01262-7

**Published:** 2020-04-19

**Authors:** Vivy T. Cusumano, Edgar Corona, Diana Partida, Liu Yang, Christine Yu, Folasade P. May

**Affiliations:** 1 Department of Medicine, David Geffen School of Medicine at UCLA, Los Angeles, California USA; 2 Vatche and Tamar Manoukian Division of Digestive Diseases, Department of Medicine, David Geffen School of Medicine at UCLA, Los Angeles, California USA; 3 Department of Gastroenterology, Southern California Permanente Medical Group, Los Angeles, California USA; 4 UCLA Kaiser Permanente Center for Health Equity, Jonsson Comprehensive Cancer Center, Los Angeles, California USA; 5 Department of Medicine, VA Greater Los Angeles Healthcare System, Los Angeles, California USA

**Keywords:** Colorectal cancer, Stool based test, Cancer screening, Prevention

## Abstract

**Background:**

The fecal immunochemical test (FIT) is the second most commonly used colorectal cancer (CRC) screening modality in the United States; yet, follow-up of abnormal FIT results with diagnostic colonoscopy is underutilized. Our objective was to determine patient-reported barriers to diagnostic colonoscopy following abnormal FIT in an academic healthcare setting.

**Methods:**

We included patients age 50–75 with an abnormal FIT result between 1/1/2015 and 10/31/2017 and no documented follow-up diagnostic colonoscopy. We abstracted demographic data from the electronic health record (EHR). Study personnel conducted telephone surveys with patients to confirm colonoscopy completion and elicit data on notification of FIT results and barriers to colonoscopy. We also provided brief verbal education about diagnostic colonoscopy. We calculated frequencies of demographic data and survey responses and compared survey responses by interest in colonoscopy after education.

**Results:**

We surveyed 67 patients. Fifty-one were aware of the abnormal FIT result, and a majority learned of the abnormal FIT result by direct communication with providers (19, 37.3%) or EHR messaging (11, 21.6%). Overall, fifty-three patients (79.1%) confirmed lack of colonoscopy, citing provider-related (19, 35.8%), patient-related (16, 30.2%), system-related (1, 1.9%), or multifactorial (17, 32.1%) reasons. Lack of knowledge of FIT result (14, 26.4%) was most common. After brief education, 20 (37.7%) patients requested colonoscopy.

**Conclusion:**

Patients with an abnormal FIT reported various multi-level barriers to diagnostic colonoscopy after abnormal FIT, including knowledge of FIT results. When provided with brief education, participants expressed interest in diagnostic colonoscopy. Future efforts will evaluate interventions to improve colonoscopy follow-up.

## Background

Colorectal cancer (CRC) is the second leading cause of cancer-related mortality in the United States (U.S.) and accounts for 8.3% of cancer deaths overall [1, 2]. The disease is largely preventable by screening, and early detection improves outcomes. Over 90% of individuals with stage I disease will survive at least 5 years, compared to less than 15% of those diagnosed at an advanced stage [3, 4]. The United States Preventative Services Task Force (USPSTF) reaffirmed the importance of CRC screening in their 2016 screening guidelines, making a grade A recommendation to screen all average-risk individuals age 50 to 75 and outlining several screening strategies. One of the recommended screening modalities, the fecal immunochemical test (FIT), is a commonly used, noninvasive, and inexpensive screening option with high participation rates [5–8]. Accordingly, the most recent Multi-Society Task Force (MSTF) CRC screening guidelines recommend FIT as one of two first-tier screening tests along with colonoscopy [9].

The FIT detects the globin portion of human hemoglobin in stool. Individuals with evidence of globin in the stool must undergo diagnostic colonoscopy to determine if there is a precancerous and/or cancerous lesion in the colon that has led to the abnormal (i.e. positive) result. While abnormal FIT results may represent benign lesions (i.e. hemorrhoids, hyperplastic polyps, normal colon), follow-up testing with colonoscopy aims to detect adenomas and colorectal carcinoma [7, 8]. Thus, FIT is a two-step screening process that is only effective when those with abnormal results undergo colonoscopy.

Despite these fundamental aspects of FIT screening, CRC screening programs struggle to achieve high rates for colonoscopic follow-up after abnormal FIT results. Across healthcare settings in the U.S., follow-up rates are far below the national benchmark of 80% colonoscopy completion after abnormal FIT [10–13]. Patients with an abnormal FIT have a CRC prevalence of 2.9 to 7.8% [8, 14]. Low follow-up rates are associated with late stage CRC at diagnosis and CRC-related death [8, 15–19]. Lack of follow-up also reduces the cost-effectiveness of screening and worsens inequities in CRC screening and CRC outcomes [20, 21].

Prior studies have identified low rates of colonoscopic follow-up after abnormal FIT and predictors of lack of follow-up [11, 12, 14, 22]. However, much of the existing literature relies on electronic health record (EHR) data to determine facilitators and barriers to colonoscopic follow-up, which is susceptible to provider bias and incomplete documentation [23]. Fewer studies have considered patient perspectives to evaluate comprehension of FIT results and to understand barriers to follow-up. Thus, we aimed to speak directly with patients in our health system with an abnormal FIT to characterize patient-reported barriers to diagnostic colonoscopy. As improving FIT follow-up rates is a priority in our health center, the work will help to identify unrecognized factors impeding FIT follow-up and guide the development of interventions to improve completion of CRC screening.

## Methods

### Study setting and population

This retrospective cohort study was conducted at University of California Los Angeles (UCLA) Health, a large, integrated academic tertiary care center in Southern California with a defined primary care population and robust referral-based care. The primary care population includes over 350,000 enrollees who exclusively receive coverage for care services at UCLA. In this setting, CRC screening and follow-up after abnormal FIT are managed by primary care providers. System-wide policy recommends CRC screening for all adults age 50 to 75, and multiple screening strategies are available with FIT (9%) and colonoscopy (85%) being the most common. If the FIT is abnormal, the primary care provider receives notification of the abnormal result and is responsible for generating a referral to gastroenterology for colonoscopy.

With assistance from our institution’s Clinical and Translational Science Institute (CTSI), we queried EHR data to identify patients who met the following inclusion criteria: 1) age 50–75, 2) assignment to a UCLA primary care provider, 3) abnormal FIT result between 1/1/2015 and 10/31/2017, and 4) lack of EHR documentation of a diagnostic colonoscopy 6 months after the abnormal FIT (as consistent with other studies in the literature) [10]. Patients were excluded if they had a history of Crohn’s disease, ulcerative colitis, prior colectomy, and/or a personal history of CRC. Patients with a family history of CRC are not screened with FIT in our health system.

### Study instrument and survey procedures

Three study personnel (V.T, E. C, and D.P) contacted all eligible patients via telephone between 8/10/18 and 9/17/18. All personnel underwent training on FIT processing and FIT guidelines prior to study initiation and participated in meetings to discuss the study protocol and survey instrument [8, 9]. Patient telephone numbers were obtained from the EHR, and study personnel attempted to reach each patient a maximum of 5 times. All patients provided verbal informed consent for participation in the study. Once verbal consent was obtained, study personnel administered a telephone survey containing 8 items. For patients that declined the survey invitation, we obtained a reason for the survey decline.

The survey instrument employed two modules to elicit data on 1) knowledge and notification of FIT results, 2) colonoscopy completion, 3) barriers to diagnostic colonoscopy, and 4) interest in colonoscopy after brief education (Table 1). Study personnel followed a script to administer the survey and to provide brief (i.e. approximately 2 min) standardized education on the purpose of a FIT test, recommendations after an abnormal result, and details of the colonoscopy procedure. The final survey items asked participants if they would like to be contacted to schedule a colonoscopy or primary care provider appointment. If participants declined, we asked the participant to report the reason for the decline. This survey instrument was informed by existing literature, reviewed by four independent study personnel, and piloted prior to use. We confirmed patient report of colonoscopy completion with EHR data.

**Table 1:** Patient survey items

	Survey Item
**Module 1: Knowledge of result, notification of result, and colonoscopy status**	
Knowledge of FIT result	“How did you learn about the result of your take home colon cancer screening stool test?”
Colonoscopy completion status	“Did you have a colonoscopy after your abnormal stool test result?”
Colonoscopy date (if completed)	“What was the date of your colonoscopy?”
Colonoscopy location (if completed)	“Where did you have the procedure done?”
**Module 2: Barriers to diagnostic colonoscopy and colonoscopy education**	
Main reason for lack of follow-up	“What would you say is the main reason why you did not have a colonoscopy after your abnormal stool test?”
Interest in colonoscopy (after brief education)	“Would it be okay for a UCLA scheduler to contact you to schedule your colonoscopy?”
Reason for not scheduling colonoscopy (if colonoscopy declined)	“Please share with me why you don’t want to schedule a colonoscopy”
Interest in primary care provider appointment (if declined colonoscopy)	“Would it be okay for a UCLA scheduler to contact you to schedule an appointment with your primary care provider?”

For each participant, we also obtained demographic data (age, sex, race, ethnicity, marital status, and insurance status at time of abnormal FIT) and method of notification of FIT result from the EHR. Six months after the survey, we performed a second manual chart review to determine whether participants completed diagnostic colonoscopy and/or a visit with their provider after participating in the survey. Findings of completed colonoscopies were also obtained from chart review.

### Statistical analysis

We first used frequencies and means (± standard deviation) to summarize participant demographic data. We then used chi-square and student t-tests to compare demographics for those that did and did not participate in the survey. Those who refused participation in the study or could not be reached by telephone were excluded from further analyses.

We next determined response frequencies for each survey item and stratified these responses by patient-reported colonoscopy completion status. For those who did not complete a colonoscopy, we calculated frequencies for the main reason for lack of colonoscopy. We also performed chi-square tests, Fisher’s exact tests, and student t-tests to compare patient characteristics between those who requested and declined colonoscopy after the brief education. All statistical analyses were performed with SAS version 9.4. A *p*-value less than 0.05 was considered statistically significant. This study was reviewed and approved by the Institutional Review Board at UCLA.

## Results

### Descriptive characteristics of the study population

**Fig. 1:**
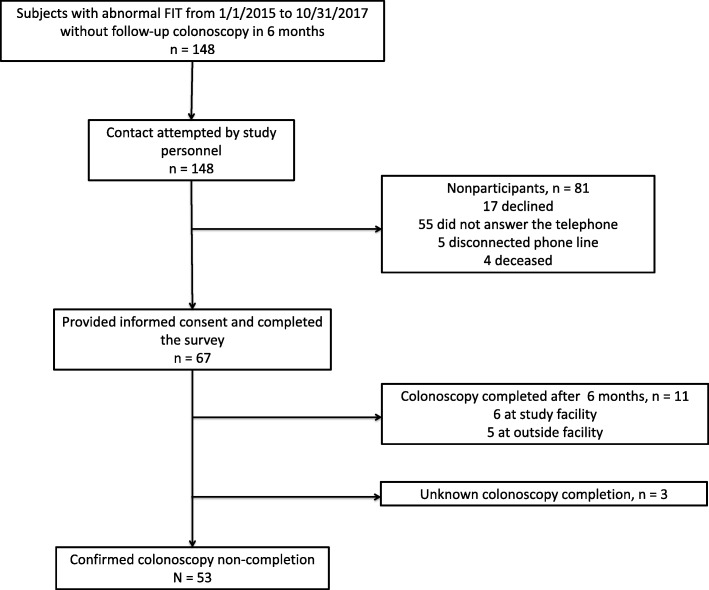
Survey participants

We contacted 148 patients, and 67 (45.3%) completed the survey (Fig. 1). For those that participated in the survey, the mean age was 63 years, 52.2% were male, and 61.2% were white. The majority of participants were married (65.7%), and 92.5% had medical insurance. The employment rate was 44.8, and 37.3% were retired (Table 2).

**Table 2:** Descriptive characteristics of survey participants and survey non-participants; *N* = 148

Patient characteristic	Survey participants (***n*** = 67)	Survey non-participants (***n*** = 81)	***p***-value*
Age (Mean ± s.d.)	63 ± 7.0	63 ± 6.9	0.58
Sex			
Male	35 (52.2%)	44 (54.3%)	0.79
Female	32 (47.8%)	37 (45.7%)	
Race/Ethnicity			
White (non-Hispanic)	41 (61.2%)	47 (58.0%)	0.20
Black (non-Hispanic)	4 (6.0%)	8 (9.9%)	
Hispanic	5 (7.5%)	0 (0.0%)	
Asian (non-Hispanic)	8 (11.9%)	10 (12.4%)	
Other (non-Hispanic)	7 (10.4%)	9 (11.1%)	
Unknown (non-Hispanic)	2 (3.0%)	7 (8.6%)	
Marital Status			
Married	44 (65.7%)	47 (58.0%)	0.40
Single	22 (32.8%)	32 (39.5%)	
Unknown	1 (1.5%)	2 (2.0%)	
Employment			
Employed	30 (44.8%)	28 (40.6%)	0.79
Unemployed	10 (14.9%)	12 (17.3%)	
Retired	25 (37.3%)	29 (42.0%)	
Unknown	2 (3.0%)	12 (14.8%)	
Medical Insurance			
Yes	62 (92.5%)	74 (91%)	0.81
No/Uncovered/Self-Pay	4 (6.0%)	7 (8.6%)	
Unknown	1 (1.5%)	0 (0.0%)	
Colonoscopy completed			
Yes	11 (16.4%)	–	–
No	53 (79.1%)		
Don’t Know	3 (4.5%)		

s.d., standard deviation

*p-values compare survey participants to non-participants

Those who did not participate in the survey (*n* = 81) did not answer the telephone (55, 67.9%), answered the telephone but declined participation (17, 20.9%), had phone numbers no longer in service (5, 6.1%), or were deceased at the time of the study (4, 4.9%). Demographic characteristics were similar among those who did and did not complete the survey (Table 2).

### Notification of FIT result and colonoscopy completion status

Of the 67 patients that participated in the survey, 51 (76.1%) were aware of their abnormal FIT result. Of these 51, most reported learning of their abnormal FIT result through direct communication with the ordering provider (19, 37.3%) or EHR messaging through the online patient-provider portal (11, 21.6%). Other mechanisms of notification included: mail (2, 3.9%), phone call by a nurse (5, 9.8%), or could not recall (14, 27.4%).

When asked about colonoscopy status, 53 of the 67 participants (79.1%) reported that they did not complete a colonoscopy after the abnormal FIT, 11 (16.4%) reported colonoscopy completion, and 3 (4.5%) were uncertain if they had a colonoscopy after the abnormal FIT. Of the 11 (16.4%) that reported colonoscopy completion after abnormal FIT, 6 did so at our health center after study initiation but prior to administration of our survey and 5 completed an outside facility colonoscopy that was not documented in the EHR.

### Reasons for lack of colonoscopy

**Fig. 2:**
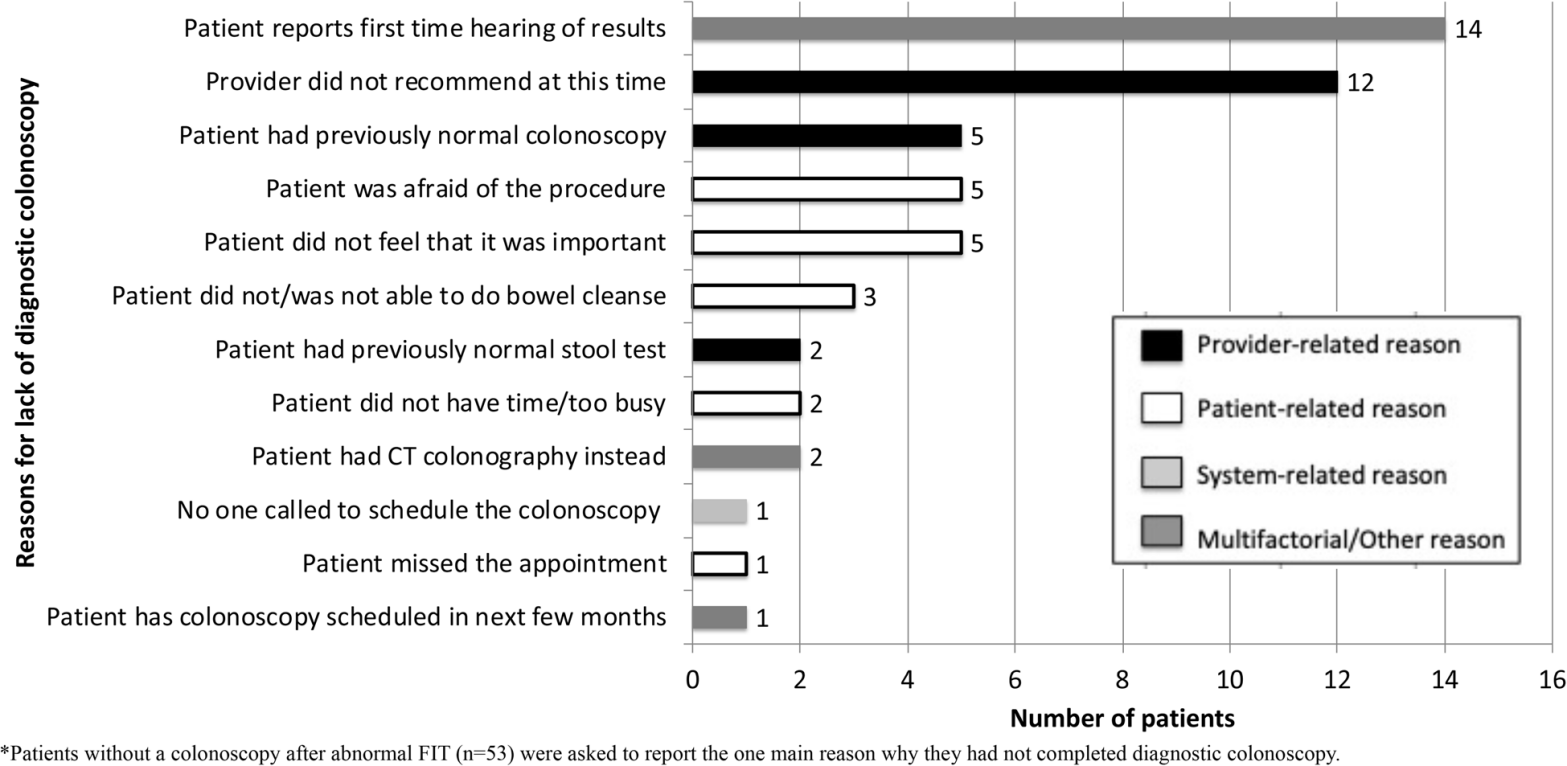
Reported main reason for lack of diagnostic colonoscopy after abnormal FIT among participants without colonoscopy* (*n* = 53)

We asked the 53 participants that did not complete colonoscopy after an abnormal FIT to state the one main reason for lack of colonoscopy. We categorized these reasons as provider-related (19, 35.8%), patient-related (16, 30.2%), system-related (1, 1.9%), or multifactorial (17, 32.1%) (Fig. 2). Patient-related reasons included patient fear and/or anxiety about colonoscopy (5, 9.4%) or believing the colonoscopy was not important (5, 9.4%). Among the provider-related reasons, primary reasons were lack of provider recommendation for follow-up colonoscopy (12, 22.6%) and provider recommendation against colonoscoy due to previous normal colonoscopy (5, 9.4%). The 1 (1.9%) participant who reported healthcare system-level barriers experienced challenges scheduling the colonoscopy procedure. There were 17 participants (32.1%) with multifactorial reasons: 14 (26.4%) reported that the survey was the first time they were made aware of their abnormal FIT result, 2 (3.8%) had a CT colonography instead of colonoscopy as the follow-up examination due to other comorbidities, and 1 (1.9%) was awaiting an upcoming scheduled colonoscopy.

Fourteen (26.4%) of the 53 survey participants that did not complete colonoscopic follow-up reported no knowlege of the abnormal FIT results at the time of the survey. Interestingly, 13 of the 14 (92.9%) did have physician or nurse EHR documentation that the abnormal FIT result was discussed with the patient.

### Interest in colonoscopy after education

After the scripted education about FIT testing and colonoscopy, 20 of 53 (37.7%) participants requested to be scheduled for colonoscopy. There were no significant differences in sociodemographic characteristics between those who did and did not want to be scheduled (Table 3). The 33 participants who declined to be scheduled for colonoscopy noted several reasons for the decline: 11 (33.3%) expressed a desire to speak to their primary care provider before proceeding with colonoscopy, 9 (27.3%) reported it was not recommended by their provider, 6 (18.2%) cited other health issues or priorities as reasons to postpone the exam, 3 (9.1%) changed locations, and 4 (12.1%) had other reasons. Chart review 6 months after the survey was administered revealed that 33 (62.3%) of the 53 patients that reported no colonoscopy at the time of the survey had further action on the abnormal FIT result after scripted education: 6 completed colonoscopy, 8 had a pending gastroenterology or colonoscopy appointment, and 19 had further discussion with their primary care provider. Of the 6 patients who completed colonoscopy, results were: normal (1), non-advanced adenoma (2), and advanced adenoma (3).

**Table 3:** Comparison of survey participants eligible for diagnostic colonoscopy stratified by interest in colonoscopy, *n* = 53

Patient characteristic	Requested colonoscopy (***n*** = 20)	Declined colonoscopy (***n*** = 33)	***p***-value
Age (Mean ± s.d.)	64 ± 7	64 ± 7	1.00
Sex			0.91
Male	10 (50.0%)	17 (51.5%)	
Female	10 (50.0%)	16 (48.5%)	
Race/Ethnicity			0.24
White	10 (50.0%)	22 (66.7%)	
Non-White	9 (45.0%)	10 (30.3%)	
Unknown	1 (5.0%)	1 (3.0%)	
Marital Status			0.85
Married	12 (60.0%)	20 (60.6%)	
Single	8 (40.0%)	12 (36.4%)	
Unknown	0 (0.0%)	1 (3.0%)	
Employment			0.28
Employed	10 (50.0%)	14 (42.5%)	
Unemployed	1 (5.0%)	7 (21.2%)	
Retired	9 (45.0%)	11 (33.3%)	
Unknown	0 (0.0%)	1 (3.0%)	
Medical Insurance			1.00
Yes	18 (90.0%)	30 (90.9%)	
No/Uncovered/Self-Pay	1 (5.0%)	3 (9.1%)	
Unknown	1 (5.0%)	0 (0.0%)	

s.d., standard deviation

*p-values compare participants who requested colonoscopy to participants who declined colonoscopy

## Discussion

Patients reported several multi-level reasons for lack of follow-up colonoscopy after abnormal FIT. These findings are consistent with studies that use EHR data to demonstrate various reasons for lack of follow-up but also highlight additional factors that play a role in poor follow-up after abnormal FIT, including lack of awareness of the FIT result and suboptimal communication around scheduling and colon preparation. Our study emphasizes the need for multi-level interventions to achieve the MSTF benchmark that 80% of patients with an abnormal FIT undergo colonoscopy [11, 12, 22, 24, 25].

We found that patients with an abnormal FIT and no colonoscopic follow-up were often unaware of the result. Lack of knowledge of the FIT result was the most common patient-reported reason for lack of follow-up, occurring in 26.4% of participants despite EHR documentation that the results were discussed with a provider for the large majority. While it is possible that providers documented a discussion that did not take place, it is also possible that patients had poor recall of discussions with providers after the abnormal FIT. This is not entirely surprising as 40-80% of medical information provided by healthcare practitioners is forgotten immediately [26, 27]. Factors that contribute to poor recall of medical information include clinician factors (use of medical terminology, amount of information relayed), mode of communication (verbal compared to written), and factors related to the patient (perceived importance, anxiety or stress, age) [27, 28]. As lack of knowledge of the abnormal result was the most common reason for lack of colonoscopy in this cohort, our findings highlight the importance of clear and purposeful information transfer and counseling regarding FIT results. The inconsistency between provider documentation and patient recall also underscores opportunities for improved tracking, navigation, and communication as patients move through the FIT screening process.

Patient- and provider-related barriers were the most common reasons for lack of colonoscopy. Patient-level barriers included fear of the procedure and the bowel preparation and low priority, which is consistent with prior literature [12, 22, 29]. Provider-related reasons included lack of provider recommendation for colonoscopy, previously normal colonoscopy, or a repeated stool screening test that was normal. They reflect provider uncertainty about appropriate use of FIT and challenges navigating discussions with patients hesistant about colonoscopy [29, 30]. The existing literature documents recent normal colonoscopy as a common reason for lack of colonoscopy after abnormal FIT or fecal occult blood test (FOBT) [14, 22]. However, the MSTF recommendation is to offer repeat colonoscopy even in the setting of a recent study, as the risk of CRC and advanced colorectal neoplasia remains notable even among those with recent colonscopy [8, 31]. These patient- and provider-related barriers are likely exacerbated by other system-related factors like the scheduling difficulties we observed. Interventions that include patient navigators to provide a targeted and individualized approach to address these emotional and structural multi-level barriers may further improve follow-up rates [25, 32]. Our very limited outreach and education about FIT and diagnostic colonoscopy generated interest and further action following abnormal FIT. However colonoscopy completion at 6 months remained low, further supporting the need for repeated patient outreach and personalized assistance to improve colonoscopic follow-up.

Our study is not without limitations. First, our study population was a majority insured patient population in a tertiary care center. Thus, our findings may not be generalizable to uninsured patients or to other healthcare settings. Nonetheless, our cohort was diverse, and the study design allowed us to focus on patient’s perceived barriers to colonoscopic follow-up beyond cost and insurance. Second, we were limited by our small sample size. However, to date, our study remains one of few to interview patients with an abnormal FIT and previous studies exploring patient perspectives are often small [29, 33]. Third, our study was limited in its scope and relies on patient recollection of events, which may be susceptible to response bias, memory bias, or poor recall. However, this limitation only highlights the need for future research to better understand challenges from the perspective of the patient and provider, especially as it pertains to communication about FIT results.

Despite these limitations, our study has many strengths. First, we directly interviewed patients to elicit their knowledge, attitudes, and beliefs about FIT screening and completing the FIT screening process. We were also able to ask patients about the main reason for lack of colonoscopic follow-up after abnormal FIT. Prior research in this area relies on chart review, in which this information is often undocumented, can be documented erroneously, or may fail to capture true patient barriers [22, 34]. Second, we highlight an area that is important and understudied. While many studies focus on CRC screening uptake, fewer explore poor follow-up after abnormal FIT despite evidence that abnormal FIT results are related to poor CRC outcomes [35, 36]. FIT screening programs are only successful if patients with abnormal results complete colonoscopy. Third, our findings improve our understanding of patient-reported barriers and provide information that will guide the development of future interventions to address barriers to colonoscopy. Lastly, we demonstrate that interest in diagnostic colonoscopy can be high, even among patients that failed to complete colonoscopic follow-up early. As a result, we are designing a telephone-based navigation intervention for patients with an abnormal FIT in our health system.

Our study has several implications for patients, providers, health care systems, and policymakers. Poor follow-up after abnormal FIT is a problem and places patients at increased risk for poor outcomes [10, 35]. Many patients are unaware of their FIT result, so providers must engage in improved counseling and repeated discussion to encourage follow-up. Clear explanation of FIT as a two-step screening process at the time of FIT prescription and addressing abnormal FIT results early may improve follow-up. Timeliness of follow-up is also critical as data suggest that follow-up more than 10 months after an abnormal FIT is associated with up to 50% higher risk of CRC and more advaned-stage disease at the time of diagnosis [18]. Concerning healthcare policy, we must increase attention nationally to these challenges in the cancer care continuum. Currently, the focus remains on CRC screening uptake, with mandated reporting of CRC screening rates through regulatory efforts like Healthcare Effectiveness Data and Information Set (HEDIS). Without a similar emphasis on completion of screening, it is unlikely that health systems will achieve high colonoscopic follow-up rates. Quality and health policy experts should consider mechanisms for reporting and monitoring rates of diagnostic colonoscopy after abnormal FIT to maximize the quality of CRC screening programs and improve CRC outcomes.

## Conclusions

Our study identified several patient-reported challenges to diagnostic colonoscopy after abnormal FIT among those without colonoscopic follow-up. Future research should include qualitative studies with patients and their providers to better characterize challenges discussing FIT results and the importance of colonoscopy. Patients might benefit from understanding upfront that abnormal FIT results require additional evaluation, and the medical community might benefit from additional education about the appropriate use of FIT, repeat FIT testing, and recommendations for colonoscopic follow-up. Given limited exisiting data, more work must also be done to inform providers of the optimal management approach for patients with an abnormal FIT and a recent normal colonoscopy. Additional research in these areas will improve FIT screening programs, enhance management and counseling of patients with abnormal FIT results, and contribute to multimodal interventions to improve colonoscopic follow-up after abnormal FIT.

## Data Availability

The datasets used during this study are available from the corresponding author upon reasonable request.

## Abbreviations

CRC: Colorectal cancer
US: United States
FIT: Fecal immunochemical test
EHR: electronic health record
MSTF: Multi-Society Task Force
USPSTF: United States Preventative Services Task Force
UCLA: University of California Los Angeles
HMO: Health Maintenance Organization
CTSI: Clinical and Translational Science Institute
HEDIS: Healthcare Effectiveness Data and Information Set
